# A Smartphone-Based Application Improves the Accuracy, Completeness, and Timeliness of Cattle Disease Reporting and Surveillance in Ethiopia

**DOI:** 10.3389/fvets.2018.00002

**Published:** 2018-01-16

**Authors:** Tariku Jibat Beyene, Fentahun Asfaw, Yitbarek Getachew, Takele Beyene Tufa, Iain Collins, Ashenafi Feyisa Beyi, Crawford W. Revie

**Affiliations:** ^1^College of Veterinary Medicine and Agriculture, Addis Ababa University, Bishoftu, Ethiopia; ^2^Department of Diagnostic Medicine and Pathobiology, College of Veterinary Medicine, Kansas State University, Manhattan, KS, United States; ^3^Cojengo Ltd, Glasgow, United Kingdom; ^4^Department of Animal Sciences, University of Florida, Gainesville, FL, United States; ^5^Department of Health Management, Atlantic Veterinary College, University of Prince Edward Island, Charlottetown, PEI, Canada

**Keywords:** cattle disease, data integrity, diagnosis, smartphone application, surveillance

## Abstract

Accurate disease reporting, ideally in near real time, is a prerequisite to detecting disease outbreaks and implementing appropriate measures for their control. This study compared the performance of the traditional paper-based approach to animal disease reporting in Ethiopia to one using an application running on smartphones. In the traditional approach, the total number of cases for each disease or syndrome was aggregated by animal species and reported to each administrative level at monthly intervals; while in the case of the smartphone application demographic information, a detailed list of presenting signs, in addition to the putative disease diagnosis were immediately available to all administrative levels *via* a Cloud-based server. While the smartphone-based approach resulted in much more timely reporting, there were delays due to limited connectivity; these ranged on average from 2 days (in well-connected areas) up to 13 days (in more rural locations). We outline the challenges that would likely be associated with any widespread rollout of a smartphone-based approach such as the one described in this study but demonstrate that in the long run the approach offers significant benefits in terms of timeliness of disease reporting, improved data integrity and greatly improved animal disease surveillance.

## Introduction

Livestock diseases affect productivity of animals through decreased yield and work output, in addition to direct mortality. In Ethiopia, livestock agriculture accounts for around 20% of the total gross domestic product, 45% of the agricultural gross domestic product (1, 2) and directly contributes to livelihood in around 65% of Ethiopian families (3). In this context, the high burden of livestock disease (4, 5) combined with limited infrastructure, poses significant challenges for animal productivity in the country (6, 7). For instance, it has been estimated that annual direct losses due to mortality account for around 8–10% of the national cattle herd, 14–16% of the sheep flock, and 11–13% of the goat flock (8).

Protecting animal and human health requires adequate disease reporting to allow appropriate action to be taken to mitigate potential risks quickly and effectively (9, 10). Surveillance systems and animal disease monitoring more generally are a major component of health-care systems (11, 12). Such systems are critical to any assessment of existing levels of disease, effectiveness of control programs and, in the context of disease eradication programs, documentation around the continued absence of disease from a given population or region, in addition to the detection of emerging diseases (11). Presence of robust animal disease surveillance systems also benefits human health as around 75% of the emerging infectious diseases that affect humans have their origin in animal populations (13).

Timely and accurate surveillance data at regional and national levels are therefore critical to support continuous improvements in animal health and in detecting outbreaks of diseases, including emerging and zoonotic diseases (14). Near real-time disease reporting, as opposed to interval-based “batch” reporting, is important in mitigating the impact of livestock disease, as early notification shortens the time between detection and the provision of effective measures for control (15). However, the current approach taken to animal surveillance in many African countries, as is the case for the Ethiopian national veterinary service department, is based on paper-based reports often prepared on a monthly basis which will inevitably only slowly reach the relevant central/national databases. In addition, these short summary reports typically indicate only total numbers of cases and lack the level of detail, such as clinical signs or disease specifics, required to estimate basic epidemiological metrics such as proportional morbidity, or the sign/disease frequencies that are critical for syndromic surveillance (16).

The application and use of smartphone technology has been more generally explored in the field of public health care (17, 18) and community-based reporting (19) within low resource settings. Such tools and services have been proposed as a means to substantially improve animal health recording, reporting, and surveillance in developing countries (12), but few detailed field-based trials have been reported in the literature. In this study, the value of a previously developed smartphone application (20), whose main aim is to assist cattle disease diagnosis, was assessed in terms of its utility for disease reporting, with the outcomes for its use in the field being compared with the traditional manual disease reporting system currently used in Ethiopia.

## Materials and Methods

The details of the smartphone app used within this study, *VetAfrica–Ethiopia*, have been described elsewhere (20). However, a brief overview of its operation may be helpful to provide context to the research discussed in this study. *VetAfrica–Ethiopia* records location and demographic data for each presenting case, together with a list of clinical signs. There is a predefined list of 16 signs from which the user is invited to indicate those that are present or absent for the case in question, after which she may indicate other signs that have been observed to be present. The Bayesian inference algorithm within *VetAfrica–Ethiopia* then uses these data to provide a diagnostic list of the potential cattle disease(s) present of decreasing likelihood. In addition to the 15 most commonly occurring diseases for cattle in Ethiopia, there is an option that “other” may be presented as part of any differential list—indicating that the clinical presentation is not higher consistent with any of the diseases being specifically evaluated within the algorithm. The user is then asked to specify which disease they believe the animal to be affected by, and in the case that they believe this to be “other” they are invited to provide the specific alternative disease. Following the clinical presentation and diagnostic steps, information is presented as to appropriate treatment options and, where relevant, details regarding any samples collected from the case for laboratory analysis are recorded.

### Study Sites and Participants

This study was conducted in 11 public veterinary clinics located in three regions of Ethiopia: Central (3 clinics), East (4 clinics), and South (4 clinics). Twelve final-year veterinary medicine students from the College of Veterinary Medicine and Agriculture of Addis Ababa University were allocated to specific veterinary clinics, as part of the Colleges’ assignment for final-year clinical practice. Six students (two in Central, two in Eastern, and two in Southern Ethiopia) were given smartphones which had the *VetAfrica–Ethiopia* application installed, while six students were similarly assigned to the other public veterinary clinics in each region but were not given smartphones, using manual diagnosis methods and paper-based recording of case data. (Two students were allocated to Bishoftu, one of the clinics in Central Ethiopia, where they worked independently but in the same clinic, one with and the other without the *VetAfrica* smartphone application.) As many of the farmers are illiterate and are not able to read and write, before approaching the case, the students asked the owner for oral consent to participate in the study mentioning that individual/personal information will not published, and then for followed by recording details of the case.

The group given Android smartphones were provided with basic training on the use of the smartphone app for clinical case management as well as instruction on how to carry out rudimentary troubleshooting, such as ensuring that recorded cases were successfully delivered to the Cloud. The selection of diseases appropriate to Ethiopia, as well as the process of app development, has been reported elsewhere (20). During the initial project information session, those students who were not chosen by random selection to work in the “smartphone” group were informed that they would be given an Android phone with the application installed at the end of the trial. The back-end services on the Cloud were delivered using Microsoft’s Azure platform, both for managing case-level data and providing access to dashboard-based data summaries. Azure provides built-in support for many important security features (https://docs.microsoft.com/en-us/azure/security/) such as encrypted data transmission and multifactor authentication, making solutions based on this platform more secure than would likely be the case if these security had been manually created by the application and/or back-end developers.

### Clinical Presentation of Cases and Proportional Morbidity by Disease

An assessment of the way in which case presentation was recorded in the traditional paper-based approach was made and compared with the case recording facilitated by *VetAfrica*. In particular, the number and form of clinical signs recorded under these alternative approaches was summarized and compared. The list of cattle diseases for cases presenting at the veterinary clinics during the study period, as diagnosed by the student practitioners using *VetAfrica* and the paper-based approach, were reviewed and enumerated. Accordingly the top ranked diseases and their estimated proportional morbidity (i.e., the relative frequency of each disease from the total cases visiting the clinics during the study period) were reported.

### Comparison of Features Recorded by *VetAfrica* to the Traditional Approach

The level of completeness associated with demographic and patient information was compared between the group using the *VetAfrica* app and the manual case recording and reporting. The time taken for case information to be reported to higher administrative levels was also compared for these two approaches. In addition, the number of clinical signs observed per case was compared. Descriptive statistics were used to explore the proportion of cases diagnosed across demographic and disease specific scenarios. Chi-square tests were used to ascertain differences in profile of sex, age, and breed by regions for both the *VetAfrica* app-assisted and manually reported cases. Potentially beneficial features of *VetAfrica* were also compared qualitatively with the manual approach, in terms of completeness, level of detail, and the mean number of days required for a report to reach higher administrative levels. The number of days required for a report to reach each level was estimated using paper records from the receiving offices (at district, zone, and federal levels) for the manual reporting approach. In the case of *VetAfrica*, the data are available to all authorized users as soon as the details of a case have been uploaded to the *Cloud* server.

Descriptive tables, statistical tests and visual summaries were prepared using libraries from the tidyverse package within R v3.1.3 (21).

## Results

### Breakdown of Cases Reported

The student practitioners who used the *VetAfrica* smartphone application and those who used the manual recording approach reported a total of 547 and 678 cases, respectively, based on cattle visiting the veterinary clinics across the three regions. A breakdown of these cases by breed, sex, and age group, according to region, can be found in Table 1. This table indicates that a relatively lower number of animals were examined in the South region, particularly for the group who used the traditional paper-based approach. The proportions of cases that related to cross and exotic bred cattle varied significantly by region, with both reporting approaches indicating much lower proportions of these breeds of cattle in the South region. In the case of the paper-based approach, there was also a significantly lower proportion of exotic cattle in the East. When considering the sex of the cases, no significant differences in proportions (*p* = 0.07) was seen in the *VetAfrica* group, but for the paper-based approach there was significantly fewer female cattle reported in the South region. In the case of cattle age, entries using the paper-based approach were inconsistent with some cattle recorded in terms of months, years, or other non-standard abbreviations; as such we aggregated according to those which could be considered to be “adult” with the rest being classified as “young” (Table 1). There were no significant differences in the proportions of those within these aggregated age groups by region, but for the case of the *VetAfrica* group there was evidence of differences in age structure across regions, with the South reporting significantly (*p* = 0.01) more diseased animals under 1 year old.

**Table 1:** Breakdown of cases in cattle refcorded using *VetAfrica* (*N* = 547) and those using traditional paper-based reporting (*N* = 678) by region and in terms of proportions across key variables within each region.

	Diagnosis and reporting using the *VetAfrica* app						Diagnosis and reporting using paper-based approach					
		Central	East	South	Sum	*p*-Value*		Central	East	South	Sum	*p*-Value*
		
	*N*	188	195	164	547		*N*	369	206	103	678	
By breed	Cross	10.1%	11.3%	4.9%	9.0%	<0.01	Cross	0.5%	5.3%	1.9%	2.2%	<0.01
	Exotic	3.7%	7.2%	0.6%	4.0%		Exotic	13.0%	1.0%	0%	7.4%	
	Local	86.2%	81.5%	94.5%	87.0%		Local	86.5%	93.7%	98.1%	90.4%	

By sex	Female	38.3%	43.1%	50.6%	43.7%	0.07	Female	50.9%	42.7%	23.3%	44.2%	<0.01
	Male	61.7%	56.9%	49.4%	56.3%		Male	49.1%	57.3%	76.7%	55.8%	

By age (months)	0–6	2.7%	4.1%	7.3%	4.6%	0.01	Young	11.7%	13.6%	7.8%	11.7%	0.32
	7–12	3.2%	2.1%	7.9%	4.2%									
	13–24	15.4%	15.9%	20.1%	17.0%									
	Over 24	78.7%	77.9%	64.6%	74.2%		Adult	88.3%	86.4%	92.2%	88.3%		

*Fisher’s exact test, young ≤2 years/24 months and adult >24 years.

### Profile of Diseases Diagnosed and Their Proportional Morbidity

**Figure 1:**
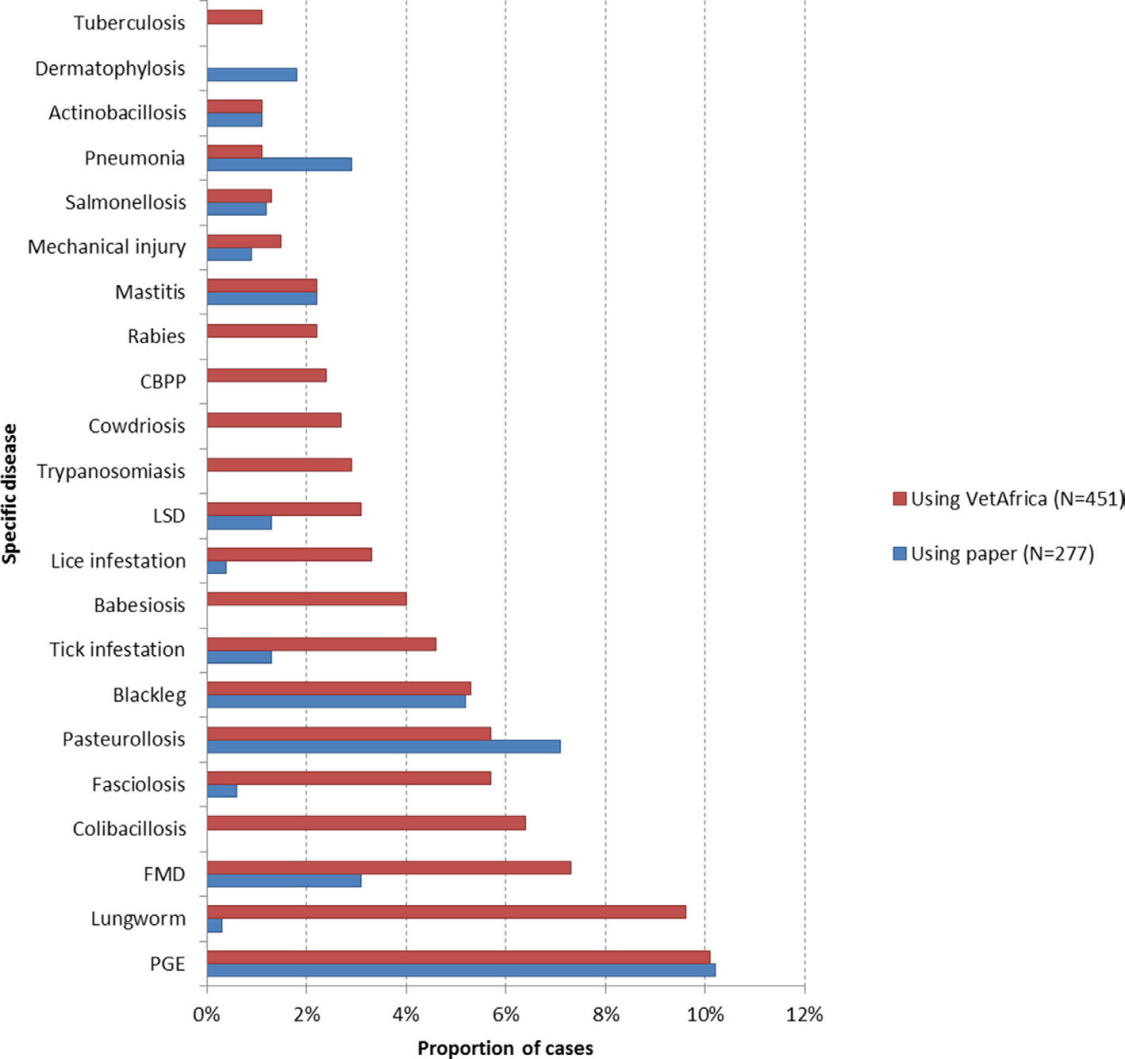
Comparison between the leading putative causes of cattle morbidity where specific disease was reported in cases using *VetAfrica–Ethiopia* (*N* = 451) and those using traditional paper-based reporting (*N* = 277). Abbreviations: PGE, parasitic gastro enteritis; FMD, foot and mouth disease; LSD, lumpy skin disease; CBPP, contagious bovine pleuropneumonia.

The profile of the top ranked diseases, as diagnosed by the student practitioners, and their respective proportional morbidity based on the two reporting approaches are given in Table 2. A graphical summary of most commonly occurring diseases, where a specific diagnosis was given, is presented in Figure 1. Common causes of morbidity in both groups were parasitic gastro enteritis (PGE), foot and mouth disease (FMD), pasteurollosis, and blackleg. Of interest is the fact that lungworm, the second most commonly reported disease by the *VetAfrica* group (~10% of all cases), was only reported twice in the cases that used manual reporting.

**Table 2:** List of most commonly occurring diseases in cattle, as diagnosed by the student practitioners, and their proportional morbidity based on the two reporting approaches.

Disease	Using *VetAfrica* (*N* = 547) (%)	Using paper (*N* = 678) (%)
Parasitic gastro enteritis^a^	10.1	10.2
Lungworm^a^	9.6	0.3
Foot and mouth disease^a^	7.3	3.1
Colibacillosis^a^	6.4	–
Fasciolosis^a^	5.7	0.6
Pasteurollosis^a^	5.7	7.1
Blackleg^a^	5.3	5.2
Tick infestation	4.6	1.3
Babesiosis^a^	4.0	–
Lice infestation	3.3	0.4
Lumpy skin disease^a^	3.1	1.3
Trypanosomiasis^a^	2.9	–
Cowdriosis^a^	2.7	–
Contagious bovine pleuropneumonia^a^	2.4	–
Rabies^a^	2.2	–
Mastitis	2.2	2.2
Dermatophylosis	–	1.8
Retained placenta	1.5	1.0
Mechanical injury	1.5	0.9
Salmonellosis	1.3	1.2
Simple indigestion	1.3	0.3
Pneumonia	1.1	2.9
Actinobacillosis	1.1	1.1
Tuberculosis^a^	1.1	–
Other	16.2	59.6

*^a^These diseases are those covered by the differential diagnostic process operating within the VetAfrica app*.

The student practitioner group which used the *VetAfrica* app diagnosed an additional 40 disease conditions (Table S1 in Supplementary Material) over and above those listed in Table 2 (which indicates all diseases that accounted for at least 1% of the proportional morbidity). This group provided a specific disease outcome for around 98% of all cases diagnosed. The group using the manual approach also provided around 40 additional diseases or “syndromes” (Table S2 in Supplementary Material). However, in just over half of cases reported by this group (51.1%), the diagnosis was given as a non-specific outcome (Table 3).

**Table 3:** Summary of main non-specific diagnoses given for cases diagnosed in cattle using the traditional paper-based approach.

Diagnosis/syndrome	Count	%	Sum (%)	Overall category
Infectious disease	95	14.0	51.1	(Non-specific diagnoses)
Infection	68	10.0		
Endo-/ectoparasite	64	9.4		
Systemic infection	46	6.8		
Septicemic	44	6.5		
Enteritis	16	2.4		
GIT problems	12	1.8		
Specific diseases	270		40.4	(As detailed in Table 2) (Infrequent diagnoses: see Table S2 in Supplementary Material)
Others (<1%)	63		8.5	

### Comparison in Terms of Reporting Completeness

Details of the information captured by those using the smartphone app and those using the paper-based approach are given in Table 4. In addition, the level of detail reported to higher administrative levels (i.e., zonal, regional, and federal level) is summarized. In the group that used the *VetAfrica* app, the full set of information captured during the diagnostic process, including each animal’s sex, age, and breed, together with a detailed list of clinical signs and the specific disease outcome were all available at any of the higher administrative levels. However, those using the manual approach only reported the total number of cases (by month) for a particular disease/syndrome, aggregated by animal species. None of the potentially valuable demographic information captured during diagnosis (each animal’s sex, age, breed, clinical signs, etc.) was transmitted to the administrative offices at the zonal, regional or federal levels.

**Table 4:** Comparison on details of information captured and reported for cattle by *VetAfrica* app users versus manual system users.

	*VetAfrica* app	Manual approach
Details captured while diagnosing (local level)	For each animal: Sex Age Breed Detailed list of clinical signs Specific disease	For each animal: Sex Age Breed Limited list of clinical signs Disease or syndrome

Details included while reporting (to higher administrative levels)	For each animal: All of the above noted data were available to all administrative levels in real time^a^	By animal species group: Number of cases (aggregated over previous month) for disease/syndrome^b^

*^a^Real-time/instant reporting depended on available Internet connection (see also next section)*.

*^b^Reported as batch updates at the end of each month*.

### Delay in Time Taken to Report

**Figure 2:**
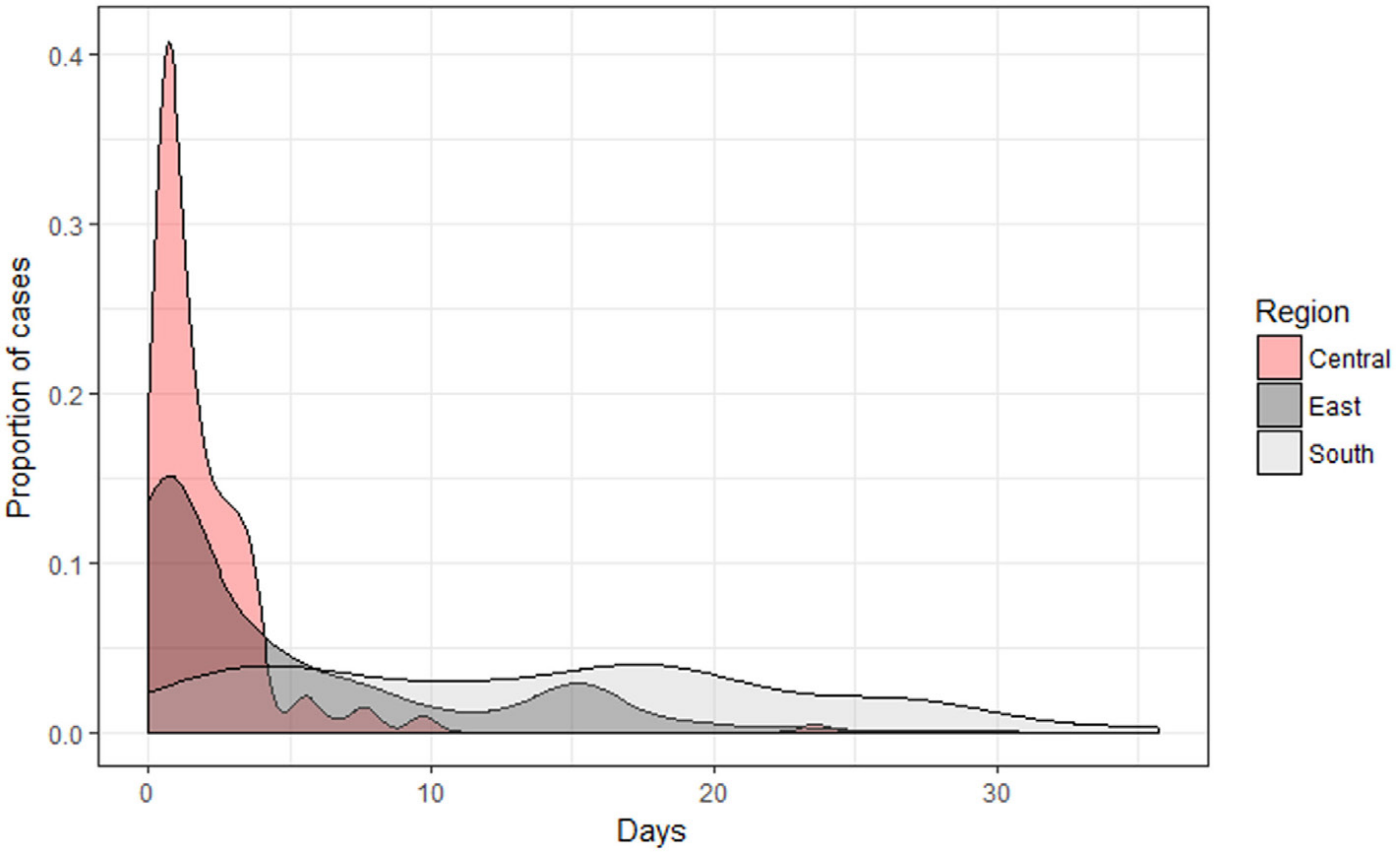
Density plot illustrating a comparison by region of the number of days required for a case to be available on the Cloud-based server when using the *VetAfrica* smartphone application.

The average times taken for case reports to become available at all administrative levels (and to all authorized access) for the group who were using the *VetAfrica* app were 2 days (95% CI: 1.6–2.3), 5 days (95% CI: 3.8–5.4), and 13 days (95% CI: 12–14.9) for the Central, East, and Southern regions, respectively. The proportions of cases reported over time are compared across the three regions in Figure 2. Cases reported from the South region took significantly longer (*p* < 0.01) when compared with the Central and East regions (up to a maximum of 35 days).

In the paper-based manual reporting approach, the chain of command states that each veterinary clinic should report the aggregated number of cases by animal species to the District Agricultural office. The District Agricultural office then sums the number of cases from the different veterinary clinics within the district and reports to the Zonal Agricultural office. This zonal office in turn aggregates the number of cases from the different districts and reports to the regional and federal veterinary offices. Although this paper-based reporting approach sounds complex and error-prone, we estimated that between 52 and 97% of the veterinary clinics reported to the district level, between 88 and 100% of the districts reported to the zone, and between 44 and 89% reported to the highest level (as shown in Table 5). Thus, for example, of the 10 veterinary clinics in the Bishoftu district reporting over a 9-month period (June 2015–March 2016), 62 (~70%) of the possible 90 clinic months had valid reports. Similarly, of the nine monthly reports expected to be reported to the zonal agricultural offices, between 4 and 8 were actually received.

**Table 5:** Proportion of returns at various levels of the reporting hierarchy (*n* = number of possible reporting months for veterinary clinics/posts in a given unit) when using the traditional paper-based approach.

Region	Reporting rates				
	District	District (from clinic)	Zone	Zone (from district)	Federal (from zone)
Central	Bishoftu	68.9% (*n* = 90)	E. Shoa	7/9	8/9^a^
	Fitche	90.5% (*n* = 63)	N. Shoa	5/9	9/9

East	Tiyo	52.2% (*n* = 63)	Arsi	7/9	NA
	Boset	90.7% (*n* = 111)	E. Shoa	8/9	(As above)^a^

South	Alem Tena	83.3% (*n* = 99)	E. Shoa	4/9	(As above)^a^
	Bora	97.7% (*n* = 36)	E. Shoa	6/9	(As above)^a^

*NA, not available*.

*^a^Four of the federal results (8/9) relate to the same zone (E. Shoa)*.

### Occurrence and Standardization of Clinical Signs

The average number of clinical signs recorded as being present for any given case by the group using the *VetAfrica* application was 5.8 (95% CI: 5.6–6.0); significantly higher than the mean of 2.2 (95% CI: 2.1–2.3) clinical signs recorded in the paper-based system. In actual fact, a number of the paper-based cases also recorded the animal’s body temperature which, given a certain threshold, could be seen as providing the additional sign of “fever”; adding these instances still resulted in a mean of less than half that seen for *VetAfrica*, at 2.5 (95% CI: 2.4–2.6) signs. It should also be noted that in many cases reported in *VetAfrica* the user indicated the absence of certain clinical signs. If the absence of a sign were also to be included then the mean number of clinical signs per case for this approach would be closer to 13. However, for the sake of making more realistic comparisons with data from the paper-based approach, we will restrict the current analyses to only those signs that were indicated to be present for a given case.

The list of clinical signs provided for cases captured using the two recording approaches is shown in Table 6. The signs are ordered according to those which occurred most frequently when signs were recorded by the group using the *VetAfrica* app. Before comparing the proportion of cases for which given signs occurred in it is important to note the “Other” at the foot of the table. This indicates that in 43% of the cases reported using the paper-based approach some sign other than one appearing on the list in Table 6 was noted. Due to wide variety of textual representation it was not possible to categorize these into groups that included more than two or three entries each, but as a whole they represented around 18% of all signs recorded (*N* = 291) using this manual approach (Table S3 in Supplementary Material). When considering signs that occurred often enough to be enumerated and compared, it is interesting to note that while “Weakness” was noted in almost three-quarters of all cases recorded in *VetAfrica*, this sign appeared in only 1% of the paper-based cases. It is likely that this “generic” sign is almost taken for granted in the manual recording system, whereas the fact that it is explicitly presented as an option in *VetAfrica* means that it is much more commonly noted. The next most commonly recorded sign in *VetAfrica* (in 70% of cases) anorexia or loss of appetite, was also by far the most commonly noted sign (in 62% of cases) in the paper-based records. However, it should be noted that while this sign was represented by a single radio button in *VetAfrica*, no less than 19 different textual representations (not including misspellings) existing for those using the paper-based approach. Some of the next most common signs were also reported by both approaches, including: weight loss, fever, diarrhea, and dyspnea (although their prevalence was higher in the case of *VetAfrica* due to the much higher number of absolute signs reporting using that approach). Certain signs, such as staring coat, anemia, lymph node enlargement, and dehydration, occurred an order of magnitude less frequently in the paper-based system; given that each of these signs appeared in at least one in five of all cases reported in *VetAfrica* it seems likely that they are being systematically and grossly underreported in the paper-based records.

**Table 6:** Proportion of times that a given sign was noted for cattle cases captured by each of the disease reporting approaches.

Sign^a^	*VetAfrica* (%)	Paper based (%)
Weakness	71	1.6
Anorexia/depression (loss of appetite)	70	62
Weight loss/emaciation (loss of body condition)	68	30
Staring coat (standing hair/rough coat)	56	8
Fever (including based on temperature reading)	44	32
Anemia and pallor	35	1.6
Diarrhea	33	11
Lymph node enlargement	25	0.1
Dyspnea/coughing (difficulty breathing)	24	15
Dehydration (x)	21	0.4
Stunted growth or pot belly	17	–
Constipation	16	0.6
Dysentery (blood in feces)	14	1.2
Submandibular/ventral edema	14	1.5
Icterus (yellowing of membranes)	12	–
Ataxia/abnormal behavior (loss of movement balance)	12	1.3
Nasal discharge (x)	10	2.4
Salivation (x)	7	7
Lameness (x)	6	5
Lacrimation (x)	3	0.7
Skin nodules (x)	3	1.0
Oral lesions (x)	3	0.4
Abdominal breathing (x)	2	2.7
Ulcer on tongue (x)	2	1.8
Mouth/teat lesions (x)	2	1.2
Hemoglobinuria (x)	2	0.3
Wound in buccal cavity (x)	1	–
Swollen forelimbs (x)	1	–
Abortion (x)	0.7	0.1
Abduction of forelimbs (x)	0.4	–
Bloating (z)	–	4.4
Crepitation (z)	–	2.5
Other	–	43

*^a^Signs that have an “x” after them indicate “optional” signs listed in the *VetAfrica* interface (i.e., they may be recorded as being observed but are not considered in the differential disease diagnostic process carried out by the *VetAfrica* app). Those followed by a “z” indicate signs that do not appear at all in any *VetAfrica* list*.

Of interest, from a *VetAfrica*, design viewpoint is the fact that all of the signs included in the main sign list appear to be useful in picking up commonly occurring signs. There appears to be evidence that dehydration should be added to the “default” list, while nasal discharge, salivation, and lameness may also be candidates. As noted above, around 18% of all paper-based signs that are noted as “Other”; signs from this set were observed in 43% of all paper-based cases (Table 6) but none of these signs was reported in more than 1% of the cases. A list of these signs is provided in Table S3 in Supplementary Material and it may be the case that a more extensive evaluation will reveal important subgroupings that could be introduced to the system.

## Discussion

In this study, we have demonstrated the potential to improve cattle disease reporting, and thus surveillance, through the use of relatively inexpensive and increasingly ubiquitous smartphone technology. Due to the real-time reporting opportunities that smartphone-based reporting and surveillance systems provide, their importance for early detection of emerging and reemerging diseases is clear. For example, during the Rift Valley Fever outbreaks of 1997 in Kenya, high rates of abortion and death of livestock likely occurred well before the human epidemic, during which over 400 people died due to Rift Valley Fever before reports of animal cases had been received at the national level (22). Smartphone-based systems can also provide details about the demography of the sick entity and clinical signs of the disease, which enhances the opportunity for early detection of unusual syndromes in the area which may be directly linked to emerging or remerging diseases. When it comes to the health of their livestock, people quickly notice unusual signs and tend to report these to health authorities, provided a working system is in operation (23). Novel approaches are also being developed to combine singles that may exist in multiple data sources associated with syndromic surveillance (24).

In this study, it was not possible to make a direct comparison with every aspect of the more traditional approach as a number of features were not available in the manual/paper-based reporting system. In the case of those aspects for which quantitative data could not be generated, an attempt was made to provide some qualitative comparisons.

Demographic data collected using the smartphone app were shown to be comparable with the data previously collected in the same regions (20). Thus, for example, we can posit that the lower proportion of cross and exotic bred animals reported in the manual recording system across the three regions when compared with those recorded in *VetAfrica* was likely due to incomplete manual reporting in the former system. As another example, the formats used when manually recording an animal’s sex or age were inconsistent, with age varying between months, years, and other non-standard abbreviated descriptors. In these cases, it seems highly likely that the *VetAfrica* derived estimates will result in more accurate aggregations and therefore in a more well-informed perspective on basic animal demography. An often reported benefit of reporting using mobile phone is the more accurate geo-referencing of case data (25). In our particular case, this was less relevant as the reports were being made from clinics whose locations were fixed and known; however, if *VetAfrica* were being used as part of a visit to cattle in their field setting, then the geo-referenced coordinates of each case could add significant value, particularly in the case of a disease outbreak where locational clustering can be a key indicator for early detection.

A major challenge of traditional reporting systems centers on the need to compile reports from various sources and provide these to central offices at regular intervals and to different administrative levels (26). The compiling process is potentially challenged by unintentional alterations of results due to errors in data submission or transcription (27). On the other hand, mobile technology or electronic based case recording and reporting systems improve timeliness, quality and interoperability of data; easing data integration (28). In addition, Robertson et al. (12) reported that such mobile phone-based surveillance system reduce the number of data entry errors and facilitate automated data analysis. The increased opportunities offered by “big data” in terms of data integration and semiautomatic analyses have been reported for both human and veterinary health data recording systems (29–31).

Those cattle diseases that have the highest importance from an economic or trade perspective were included in the *VetAfrica–Ethiopia* app, based on the diseases targeted for control by the veterinary services of Ethiopia (4). These diseases were seen to have relatively high levels of proportional morbidity based on both the *VetAfrica*-assisted and manual reporting approaches. However, in the manual system, almost 60% of case reports did not provide a specific disease outcome; they simply noted non-specific signs or syndromes. For instance, the common causes of morbidity as recorded in both manual and VAE-assisted diagnoses were PGE, FMD, pasteurollosis, and blackleg. Lungworm was only reported twice in the cases that used manual reporting while it is the second most commonly reported disease by the *VetAfrica* group (~10% of all cases). It may well be that such cases were simply reported as “systemic infection” or “infectious disease” when using the paper-based approach. Some of the specific diseases (such as PGE, salmonellosis, pneumonia, and mastitis) reported using the paper-based approach by some clinicians might be registered as more generic GIT problems, enteritis, bacterial infections, systemic infection, or an infectious disease. These non-specific outcomes make disease aggregation and estimation problematic and may mask the importance of certain economically important diseases. Even gaining estimates of simple proportional morbidity within cattle populations is difficult based on this type of data. Despite this, these non-specific signs and syndromes may have some utility in the context of syndromic surveillance (32–35).

In a *Guideline for Evaluating Public Health Surveillance Systems* (36), completeness and timeliness are identified as being crucial measures of surveillance data quality. We found that the manual reporting approach omitted valuable demographic information as well as details relating to disease and clinical signs when reporting to higher administrative levels. This is not uncommon in low resource settings. In a recent survey of animal health care in rural Uganda, community animal health workers noted that only in around 10% of reports did they specify “disease identified,” with “number of sick/treated animals” being the much more common surveillance metric (37). These data are expensive to collect (requiring the veterinarian’s time as well as the cost of stationery) and often cannot be used at higher administrative levels for further analysis, such as for the syndromic categorization of clinical signs. Furthermore, we found that in some instances almost 50% of cases recorded at the clinic level were never reported to the district agricultural office and that this number could deteriorate further when reporting to higher levels. This has obvious implications in terms of the inconsistency in reporting from different clinics, districts, and zones. It can be seen that the regions like the South had the poorest level of reporting, probably due to the fact that this region is physically distant from the federal veterinary office. which is based in Addis Ababa. By contrast, in the case of *VetAfrica*-assisted reporting, every case was available for review/analysis as all levels of administration through access to data on a secure Cloud-based server. Such infrastructural efficiencies and ubiquitous access have been at the core of some of the most innovative uses of these technologies in the sphere of human medicine, with the Global Trachoma Mapping Project providing a particularly impressive example in a number of low and middle income country settings (38). It was, however, noted that significantly longer delays occurred when reporting cases from very remote rural areas. This challenges the notion that mobile Internet technology can lead to instantaneous reporting from any animal health worker in possession of a smartphone, as telecommunications infrastructure may remain a limiting factor in such remote locations. In this study, we observed that *VetAfrica*-assisted reporting from the South region took significantly longer time when compared with the Central and East regions which is likely due to the fact that more remote areas tend to have less reliable access to the Internet and/or mobile data services.

Ideally, the accuracy of the data collected by such applications should be supported by field evaluation as to disease outcomes for all cases. In our study, it was not possible to conduct field evaluation using laboratory confirmation due to logistic constraints including cold chain to keep the samples to destined locations, shortage of laboratory consumables, processing costs, etc. These constraints appear to be shared by many studies of surveillance systems (39, 40); one of which noted that only 17 out of 221 systems assessed included such evaluation. Indeed, Walker (26) notes that one of the primary barriers to implementation of new methods in veterinary and human surveillance has been the lack of evaluation of such systems.

To the best of our knowledge, this is the first attempt to carry out a full field-based trial that addresses both cattle disease diagnosis and reporting using smartphones in a resource-limited setting. Even within human medicine, a number of the best known examples have adopted a text messaging approach, given that these can be implemented using feature phones (41, 42). A recent survey exploring the potential for mobile phone use to deliver animal health information in Uganda found that while almost all livestock keepers owned a feature phone, only around 10% owned a smartphone (43). However, based on recent trends, the shift toward smartphones and more fully functional apps is likely to continue at pace (44). Such mobile apps provide clear benefits not only in comparison to manual paper-based data collection and reporting but also to simple SMS-based approaches, in terms of gathering more consistent and complete demographic and epidemiological information. Approaches such as that demonstrated by *VetAfrica* offer opportunities for improvements in disease reporting and surveillance within developing countries and can facilitate the early detection of emerging diseases. While such smartphone-assisted reporting and surveillance can present considerable start-up challenges in terms of financial resources and sporadic mobile network coverage; in the long run, they offer clear benefits in terms of timeliness, improved data integrity, and ultimately a reduction in operating costs.

## Ethics Statement

This work did not require neither sampling nor experimentation on animals or humans. However, informed consent was taken from animal owners during data collection. In addition, the handling of animals was overseen by the appropriately qualified veterinarian who was supervising the final year students as part of their training, under the normal procedures laid out in the College of Veterinary Medicine clinical rotation guidelines of Addis Ababa University.

## Author Contributions

CR and IC conceived the idea of the study, developed the smartphone app, and trained the group in its use. TB, TT, IC, AB, and CR designed the fieldwork, organized the data downloaded from the cloud server, analyzed the data, interpreted results, wrote the manuscript, and approved the version to be published. FA and YG helped in the data collection, in drafting the manuscript and approved the final version of the manuscript.

## Conflict of Interest Statement

The authors declare that the research was conducted in the absence of any commercial or financial relationships that could be construed as a potential conflict of interest. IC was at the time of the study acting as Technical Director for Cojengo, the software development partner in this IDRC-funded project. The reviewer AA and handling editor declared their shared affiliation.
