# Supplementary material for: A Smartphone-Based Application Improves the Accuracy, Completeness, and Timeliness of Cattle Disease Reporting and Surveillance in Ethiopia
Source: Front Vet Sci. 2018 Jan 16;5:2. doi: 10.3389/fvets.2018.00002 (PMC5776010; doi:10.3389/fvets.2018.00002)
Supplement: Supplementary file 1 [file Table_1.PDF]

Supplementary Information

For article in *Frontiers in Veterinary Science* - "A smartphone-based application improves the accuracy, completeness and timeliness of cattle disease reporting and surveillance in Ethiopia" (Beyene *et al*, 2017).

Table S.1: List of infrequently occurring disease conditions (with number of occurrences) as reported by those using the *VetAfrica* app.

**Disease/Condition**	**# cases**
abscess	1
acidosis	5
actinomycosis	2
aspergilloses	1
bloat	3
botulism	3
bovine farcy	1
brucellosis	2
coccidiosis	1
conjunctivitis	1
dermatophilosis	1
dermatophytosis	2
endoparasite	3
epistaxis	1
oesophageal choke	2
oestrus cycle	2
ectoparasite	1
flea Infestation	1
food poisoning	1
grain Overload	4
haemonchosis	2
hernia	2
lameness	1
leech Infection	3
listeriosis	1
malignant catarrhal fever	1
malignant oedema	1
mange mite	1
oedema	1
otitis	1
parafilaria	1
paraphimosis	1
paratuberculosis	3
photosynthesization	2
pregnancy diagnosis	1
septicaemia	2
systemic disease	4
urolithiasis	2
vaginal prolapse	1
