# Supplementary material for: A Smartphone-Based Application Improves the Accuracy, Completeness, and Timeliness of Cattle Disease Reporting and Surveillance in Ethiopia
Source: Front Vet Sci. 2018 Jan 16;5:2. doi: 10.3389/fvets.2018.00002 (PMC5776010; doi:10.3389/fvets.2018.00002)
Supplement: Supplementary file 2 [file Table_2.PDF]

Supplementary Information

For article in *Frontiers in Veterinary Science* - "A smartphone-based application improves the accuracy, completeness and timeliness of cattle disease reporting and surveillance in Ethiopia" (Beyene *et al*, 2017).

Table S.2: List of infrequently occurring disease conditions (with number of occurrences) as reported by those using the paper-based system.

Disease/Condition	# cases
actinomycosis	1
allergy	2
bloating	2
brisket oedema	1
brucellosis	1
castration	1
conjunctivitis	1
constipation	2
demodicosis	1
dermatitis	1
dermatophytosis	2
dystocia	1
feed overload	1
fungal infection	1
grain overload	2
hematoma	1
hypocalcaemia	1
infertility	2
leech	1
local abscessation	1
mineral deficiency	1
oesophageal obstruction	1
paraphimosis	2
paratuberculosis	2
pregnancy toxaemia	1
requested for feedlot	1
respiratory disease	3
septicaemic pasteurollosis	1
shipping fever	1
swelling	4
thelaziosis	2
toxicosis	1
trauma	1
urinary system infection	1
uterine infection	2
viral infection	1
white scours	2
wound	3
wound inside the mouth	1
