# Supplementary material for: A Smartphone-Based Application Improves the Accuracy, Completeness, and Timeliness of Cattle Disease Reporting and Surveillance in Ethiopia
Source: Front Vet Sci. 2018 Jan 16;5:2. doi: 10.3389/fvets.2018.00002 (PMC5776010; doi:10.3389/fvets.2018.00002)
Supplement: Supplementary file 3 [file Table_3.PDF]

Table S.3: Infrequently reported signs by those using the paper-based system to report cases. In total these accounted for around 18% of all signs recorded (N = 291 out of 1679 signs), but none of them occurred in >1% of cases.

7 days ago heavily kicked	history of eating grain	swelling of jaw
abdominal swelling	inflammation of the body	swelling of penis
abnormal milk	inflammation of the legs	swelling of prepuce
abscess on the neck	injury of the head by a bull	swelling on both sides, prescapular area
aggressive behavior	itching the skin	swelling on hind limb and rupture
allergy	kicking the ground	swelling on lower part of the belly
alopecia	lesion on the leg and hoof	swelling on the abdomen
anthriftiness	lice and tick infestation	swelling on abdomen and tip of udder
behavioral change	local wound on hind limb with pus	swelling on the back area
bleeding nose	louse on its body	swelling on back region with discharge
bleeding on its tail	mucoid faeces	swelling on gluteal muscle and licking
bleeding on the neck	nausea	swelling on the head & leg region
bleeding while milking	nodules	swelling on the hind leg
blood mixed milk	obstruction of the rumen	swelling on the hoof
bloody discharge from mouth	offensive discharge/ no pregnancy	swelling on the leg with pus
bottle under neck	offensive discharge using speculum	swelling with pus discharge
bottled jaw	one eye protruded	swellings on all body parts of the animal
broken horn	pain to touch	swollen throat
coagulated milk	presence of tick	tachycardia
abdominal breathing	rapid breathing	teeth grinding
colic	restless	teat of cow is not letting down milk
congested mucus membrane	retained placenta	tick infestation
congestion	roaring sound	tick/flea infestation
decreased milk production	ruminal stasis	trauma on the right hind leg
deep and wide wound	rupture and bleeding of the skin	udder swelling (hot and painful)
difficulty defecating	scratching on standing objects	inability of pregnancy
difficulty of giving birth	shivering	unable to drink water
difficulty of urination	sign of giving birth	unable to ingest feed when feeding
dilation of the eye	skin irritation	unable to release the faeces
dirty body	skin lesion	unable to urinate
discharge from mouth	slight swelling on the skin	unable to walk
discharge, non-specific	small wounds on digital extremities	uncoordinated movement
discoloration of the eye	sneezing	unthriftiness
discomfort during urination	standing recumbence	white greyish pus from the swelling
dropping head	straining	whitish ear
discharge from the mouth	stretching	wound around prescapular area of skin
dry faeces	stretching neck	wound at the neck region
dry muzzle	swelling of the lower jaw	wound infection
ectoparasite	swelled glans penis	wound-like lesions around mouth area
fear of light	swelling around the masseter muscle	wound on the digital extremities
fertility problem	swelling around the ribs	wound on the foot root
grunting	swelling around tracheal area	wound on the forelimb
hairless dead calf	swelling of belly	wound on the gluteal muscle
haemorrhage	swelling of both ears	wounds on different parts of the body
haemorrhagic mucus membrane	swelling of dewlap	yellowish milk
